# Strong magnetophonon oscillations in extra-large graphene

**DOI:** 10.1038/s41467-019-11379-3

**Published:** 2019-07-26

**Authors:** P. Kumaravadivel, M. T. Greenaway, D. Perello, A. Berdyugin, J. Birkbeck, J. Wengraf, S. Liu, J. H. Edgar, A. K. Geim, L. Eaves, R. Krishna Kumar

**Affiliations:** 1 School of Physics & Astronomy, University of Manchester, Manchester, M13 9PL UK; 2 National Graphene Institute, University of Manchester, Manchester, M13 9PL UK; 3 Department of Physics, Loughborough University, Loughborough, LE11 3TU UK; 4 School of Physics & Astronomy, University of Nottingham, Nottingham, NG7 2RD UK; 5 Department of Physics, University of Lancaster, Lancaster, LA1 4YW UK; 6 Department of Chemical Engineering, Kansas State University, Manhattan, KS 66506 USA

**Keywords:** Graphene, Electronic properties and materials, Electronics, photonics and device physics

## Abstract

Van der Waals materials and their heterostructures offer a versatile platform for studying a variety of quantum transport phenomena due to their unique crystalline properties and the exceptional ability in tuning their electronic spectrum. However, most experiments are limited to devices that have lateral dimensions of only a few micrometres. Here, we perform magnetotransport measurements on graphene/hexagonal boron-nitride Hall bars and show that wider devices reveal additional quantum effects. In devices wider than ten micrometres we observe distinct magnetoresistance oscillations that are caused by resonant scattering of Landau-quantised Dirac electrons by acoustic phonons in graphene. The study allows us to accurately determine graphene’s low energy phonon dispersion curves and shows that transverse acoustic modes cause most of phonon scattering. Our work highlights the crucial importance of device width when probing quantum effects and also demonstrates a precise, spectroscopic method for studying electron-phonon interactions in van der Waals heterostructures.

## Abstract (web summary)

Increasing the size of mesoscopic devices based on van der Waals heterostructures triggers additional quantum effects. Here, the authors observe distinct magnetoresistance oscillations in graphene/h-BN Hall bars only in devices wider than 10 μm due to resonant scattering of charge carriers by transverse acoustic phonons in graphene.

## Introduction

Two-dimensional electronic systems exhibit a rich variety of quantum phenomena^1,2^. The advent of graphene has not only provided a way to study these phenomena in the quasi-relativistic spectrum, but has also extended their experimental range^3,4^, made some observations much clearer^5–8^ and, of course, revealed many more effects^9–12^. These advances are mostly due to graphene’s intrinsically high carrier mobility that is preserved by state-of-the-art heterostructure engineering in which graphene is encapsulated between hexagonal boron nitride layers^13,14^ and electrically tuned with atomically smooth metallic gates^8,15^. Nonetheless, one of the first discoveries in quantum transport, well known for over 50 years^16,17^, has remained conspicuously absent in graphene–magnetophonon oscillations^18,19^.

**Fig. 1:**
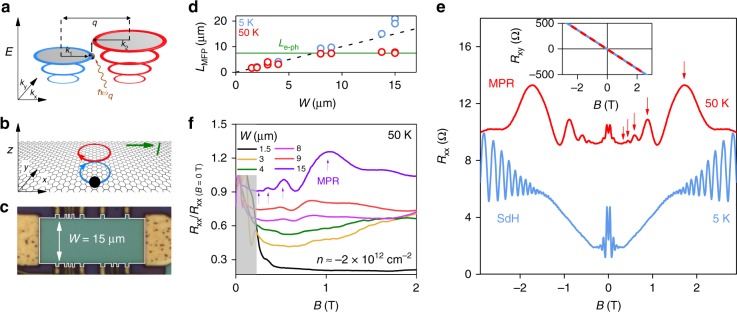
Size-dependent magnetoresistance oscillations in mesoscopic graphene devices. **a** Landau level spectra of graphene. The diagram illustrates a carrier with momentum *k*_1_ (black sphere) making a transition between Landau levels (blue and red rings) by resonant absorption of a phonon (brown arrow) with momentum *q* = |***k***_1_−***k***_2_| and energy *ħω*_*q*_. Solid black arrows represent the magnitudes of wave vectors *k*_1_, *k*_2_ and *q*. **b** The semiclassical motion of a carrier (black sphere) in real space for the resonance condition sketched in **a**. The red and blue circles which touch at a tangent point represent the initial and final semiclassical cyclotron orbits of two Landau-quantised states between which an electron is scattered by a phonon. During resonant scattering, the carriers follow a path that resembles the number 8 (figure of eight trajectory). The red and blue arrows show the motion of the charge carriers along this trajectory. The green arrow indicates direction of the applied current *I*. **c** Optical image of a graphene device with *W* = 15 μm. The edges of the mesa are indicated by the white solid line. **d** Open circles plot experimentally determined Drude mean free path *L*_MFP_ as a function of *W* for two *T* and fixed *n* = −2 × 10^12 ^cm^−2^. Black dashed line plots the equation *L*_MFP_ = *W*. Solid green line marks the phonon-limited mean free path (*L*_e–ph_) at 50 K. Our measurements focussed on the valence band because our wide devices exhibited higher electronic quality for hole doping (**e**), longitudinal magnetoresistance data *R*_*xx*_ (*B*) for fixed *n* = −3.3 × 10^12 ^cm^−2^ measured in our wide device (**c**) at two different *T*. The red arrows indicate peaks that are caused by magnetophonon resonance (MPR). The 50 K curve is off-set vertically for clarity. Inset: Hall resistance *R*_*xy*_(*B*) measured simultaneously as *R*_*xx*_. The solid blue and dashed red lines are data measured at 5 and 50 K respectively. **f** *R*_*xx*_/*R*_*xx* (*B* = 0T)_ measured at fixed *n* and *T* in several devices of different *W*. The shaded area close to *B* *=* 0 contains semiclassical effects^4^

In the presence of an applied magnetic field (*B*), electrons in pristine crystals become localised in closed orbits and their spectra take the form of quantised Landau levels (LLs) separated by energy gaps. However, an electrical current can still flow in the bulk due to carriers resonantly scattering between neighbouring orbits by the absorption or emission of phonons with energies equal to the LL spacing^19^ (Fig. 1a). In a semiclassical model, the resonant transitions occur between orbits which just touch in real space and induce figure of eight trajectories^20^ (Fig. 1b), corresponding quantum mechanically to strong overlap of the tails of their wave functions in the vicinity of their classical turning point. This effect, known as magnetophonon resonance (MPR) causes magnetoresistance oscillations that are periodic in inverse magnetic field^19,21^. Whereas magnetophonon oscillations have been used extensively for studying carrier–phonon interactions in bulk Si and Ge^22^, semiconducting alloys^18^ and heterostructures^23–25^, there have been no reported observations in any van der Waals crystal, not even graphene, despite its exceptional electronic quality.

In this article, we consider a subtle yet crucial aspect concerning the design of electronic devices based on graphene, namely the lateral size of the conducting channel. It has so far remained small, only a few micrometres in most quantum transport experiments. Our measurements using graphene Hall bars of different widths show that wider samples start exhibiting pronounced magnetophonon oscillations.

## Results

### Phonon scattering in wide graphene channels

Our experiments involved magnetotransport measurements on graphene Hall bars encapsulated by hexagonal boron nitride, with particular attention paid to ‘wide’ devices with channel widths *W* > 10 μm. An optical image of one of our widest devices is shown in Fig. 1c (see Supplementary Note 1 for details of device fabrication). Because the electron–phonon coupling is so weak in graphene^26^, charge carriers scatter more frequently at the device edges of micron-sized samples rather than with phonons in the bulk, especially at low temperature^27^ (*T*). This is evident when comparing the Drude mean free path (*L*_MFP_) for devices of different *W* and a fixed carrier density (*n*) of holes (Fig. 1d). At 5 K, all devices exhibit size-limited mobility (*L*_MFP_ > *W*) because carriers propagate ballistically until they collide with the edges of the conducting channel. Even at 50 K, scattering is still dominated by the edges in most of our devices and *L*_MFP_ increases linearly with *W*. However, at these higher temperatures we find that *L*_MFP_ saturates around 8 μm (green line in Fig. 1d) and does not increase upon further widening of the device channel. This saturating behaviour tells us that *L*_MFP_ is no longer dependent on the device width and carriers scatter mostly with phonons in the bulk (*L*_MFP_ < *W*). In effect, widening the channel makes our measurement more sensitive to bulk phenomena rather than edge effects.

### Width-dependent magnetoresistance oscillations

The main observation of our work is presented in Fig. 1e, which plots the longitudinal resistance (*R*_*xx*_) of a 15 μm wide Hall bar (Fig. 1c) as a function of *B*, at two *T* and fixed *n*. At 5 K we observe two distinct oscillatory features. The first, at relatively low *B* < 0.2 T, are the well-established semiclassical geometrical resonances that occur due to magnetic focussing of carriers between current and voltage probes^4^ (see Supplementary Note 2, Supplementary Fig. 2). At higher *B* (∼1T), quantised cyclotron orbits are formed and we observe 1/*B*-periodic Shubnikov de Haas (SdH) oscillations. Their origin is confirmed by noting that the charge carrier density *n* = 4*e*/(*h*Δ(*B*^−1^)) extracted from the SdH period (Δ(*B*^−1^)) agrees with that determined by Hall effect measurements (Fig. 1e, inset). At 50 K, the low-field geometric oscillations remain visible although their relative amplitudes are suppressed due to the reduced carrier mean free path. However, at higher |*B*| *>* 0.2T, an additional set of oscillations appears with five clear maxima (indicated by red arrows in Fig. 1e). These high *T* oscillations are also periodic in 1/*B* but are distinguished by their markedly slower period. In contrast to *R*_*xx*_, the Hall resistance, *R*_*xy*_, shows no oscillatory features and has the same value at both *T* (Fig. 1e, inset), confirming that *n* does not change upon warming the sample.

The observation of the high *T* oscillations depends critically on the sample width. This is shown in Fig. 1f which plots the normalised magnetoresistance, *R*_*xx*_/*R*_*xx* (*B* = 0T)_, for devices with different *W* at fixed *T* and *n*. We note that the bulk channels in all our devices are intrinsically clean and free from defects (probed by ballistic transport experiments in Supplementary Note 3, Supplementary Fig. 3). Nonetheless, whereas these oscillations are well developed in the widest devices (resonances marked by purple arrows), they are poorly resolved for devices with *W* < 8 μm and completely absent in the narrowest one (*W* = 1.5 μm). As described below, we identify these size dependent, high *T* oscillations with MPR

**Fig. 2:**
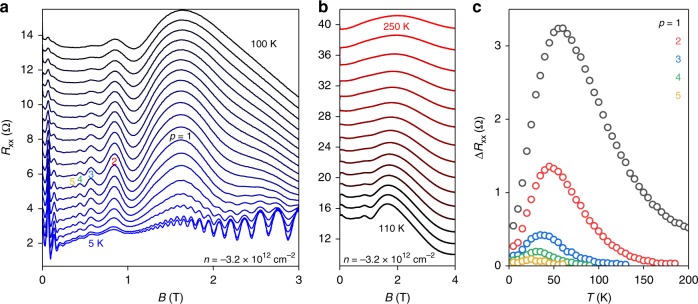
Temperature dependence of the magnetophonon effect. **a** Magnetoresistance *R*_*xx*_ (*B*) for *T* between 5 K (blue curve) and 100 K (black curve) in 5 K steps for fixed *n* measured in another Hall bar with *W* = 15 μm. **b** Extended data set of **a** showing high *T* behaviour (10 K steps). **c** Temperature dependence of the amplitude of MPR peaks, Δ*R*_*xx*_ (*T*), indicated in **a** by colour coded letters, *p* = 1–5

A defining feature of magnetophonon oscillations is their unique non-monotonic temperature dependence, in which their amplitude first increases with *T* and then decays^25^. Figure 2a shows the temperature dependence of *R*_*xx*_ (*B*) for fixed *n* between 5 and 100 K (5 K steps) for another wide Hall bar device (*W* = 15 μm). In this sample, weak magnetophonon oscillations already appear at 5 K in the field range between the geometric and the SdH oscillations. The resonances are labelled *p* = 1 to 5, where the integer *p* refers to the number of LL spacings that are crossed during the transition; *p* = 1 corresponds to scattering between LLs adjacent in energy (Fig. 1a). With increasing *T*, the magnetophonon oscillations become more pronounced as more phonons are thermally activated, while the SdH oscillations are strongly suppressed. Although both phenomena require carriers that exhibit coherent cyclotron orbits (*μB* > 1, where *μ* is the carrier mobility), MPR is not obscured by smearing of the Fermi–Dirac distribution across Landau gaps^25^; rather it is enhanced due to an increased number of unoccupied states into which carriers can scatter. Hence magnetophonon oscillations persist to higher *T* than SdH oscillations. However, they are eventually damped at high enough *T* (Fig. 2b) when LLs become broadened by additional scattering (*μB* ∼ 1). This non-monotonic behaviour is better visualised in Fig. 2c which plots the oscillatory amplitudes (Δ*R*_*xx*_) as a function of *T*. Notably, the amplitude of all resonances peak at *T* below 60 K, corresponding to a thermal energy of a few meV.

### MPR spectroscopy

**Fig. 3:**
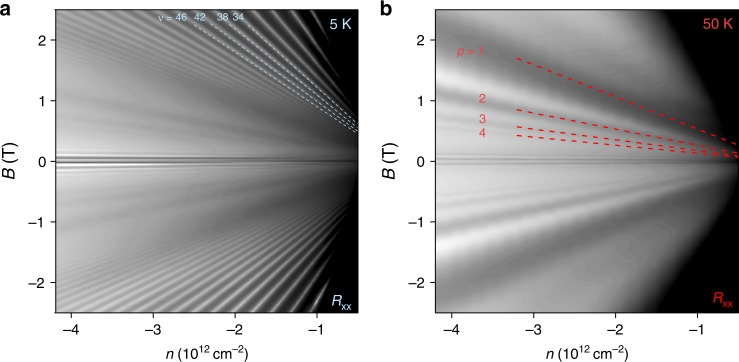
Density dependence of magnetophonon oscillations. **a** Longitudinal resistance *R*_*xx*_ (grey scale map) as a function of *n* and *B* measured at 5 K (*W* = 15 μm). Logarithmic grey scale: white: 1 Ω to black: 15 Ω. The blue dashed lines trace Landau gaps corresponding to high filling factors ν = *nh*/*Be*. **b** Same as **a** measured at 50 K. Logarithmic grey scale: white: 5.5 Ω to black: 18 Ω. The red dashed lines plot Eq. (1) for *p* = 1 to 4 which corresponds to carriers scattering with transitions across 1 to 4 Landau level spacings. Features appearing for *B* < 0.2 T are the semiclassical geometrical oscillations^4^ not relevant in this work (see Supplementary Note 2 for details)

For the doping levels and *B*-fields at which the oscillations occur, the charge carriers occupy high-index LLs (*N* ∼ 20 for *p* = 1) separated by small energy gaps (∼5 meV) with a classical cyclotron radius up to *R*_c_ ∼ *ħk*_F_/*eB* ∼ 300 nm, where *k*_F_ is the Fermi-wave vector. Resonant inter-LL transitions occur due to inelastic scattering by low-energy acoustic phonons that induce figure of eight trajectories (Fig. 1b). This type of trajectory occurs with high probability because the wave functions of the initial (blue circle in Fig. 1b) and final states (red circle) have a large spatial overlap where they touch in real space^24^. During figure of eight trajectories, the velocity of the carrier is reversed at the intersection of the two cyclotron orbits (see arrows in Fig. 1b). This process requires a phonon of specific momentum *q* ≈ *2k*_F_ ∼ 10^9^ m^−1^ and energy *ħω*_*q*_ (2*k*_F_) ∼5 meV that can back-scatter the carriers during the inter-LL transition. Energy and momentum conservation for such a process requires that *E*_*N*+p_ − *E*_*N*_ = *ħω*_*q*_ (2*k*_F_), where *E*_*N*_ is the energy of an electron in the *N*th LL, so that resonances occur at *B* values given by

1 $$B_{p} = \frac{{nhv_{\mathrm{s}}}}{{pev_{\mathrm{F}}}}$$

(see Supplementary Note 4 for a detailed derivation using the semiclassical model). Here, *v*_F_ and *v*_s_ are the Fermi velocity and low-energy acoustic phonon velocity in graphene respectively. This resonant condition is unique to massless Dirac electrons and is strikingly different to the case of massive electrons in a conventional two-dimensional electron gas (2DEG) system^24^ where *B*_p_ scales with *n*^0.5^. On resonance, inelastic scattering between neighbouring orbits (Fig. 1b) gives rise to a finite and dissipative current in the bulk. This behaviour causes maxima in *ρ*_*xx*_ at *B*_*p*_; the 1/*B* periodicity results in oscillations described by Δ*ρ*_*xx*_ ∼ e^−*γ/B*^cos(2*πB*_F_*/B*) where *B*_F_ ≡ *pB*_p_ and the factor *γ* depends on temperature^28^. Equation (1) predicts that the position of maxima scales linearly with *n*. With this in mind, Fig. 3a, b plot maps of *R*_*xx*_ (*n*, *B*) for one of our 15 μm devices at 5 K (Fig. 3a) and 50 K (Fig. 3b). In addition to the typical Landau fan structure that is dominant at low *T* (filling factors, ν, are marked by blue dashed lines), the maps reveal a broader set of fans at lower *B* that are more prominent at 50 K (Fig. 3b). They are caused by MPR (*p* values are labelled in red) and demonstrate that their frequency scales linearly with *n*. Furthermore, the positions of MPR peaks in Fig. 3b can be fitted precisely by Eq. (1) (red dashed lines) with a constant *v*_s_/*v*_F_ = 0.0128. By studying the temperature dependence of SdH oscillations in our graphene devices (Supplementary Note 5, Supplementary Fig. 4), we extract *v*_F_ and determine *v*_s_ accordingly. We note that *v*_F_ shows no significant dependence on *n*, as expected for graphene devices on hexagonal boron nitride at high doping^29^, because e–e interactions that cause velocity renormalisation^30^ are heavily screened. Hence, using the extracted *v*_F_ = 1.06 + 0.05 × 10^6 ^ms^−1^, we determined a phonon velocity, *v*_s_ = 13.6 + 0.7 km s^−1^. This value is close to the speed of transverse acoustic (TA) phonons in graphene (∼13 km s^−1^) calculated in numerous theoretical works^31–35^. Therefore, we infer our oscillations arise from inter-LL scattering by low energy and linearly dispersed TA phonons.

**Fig. 4:**
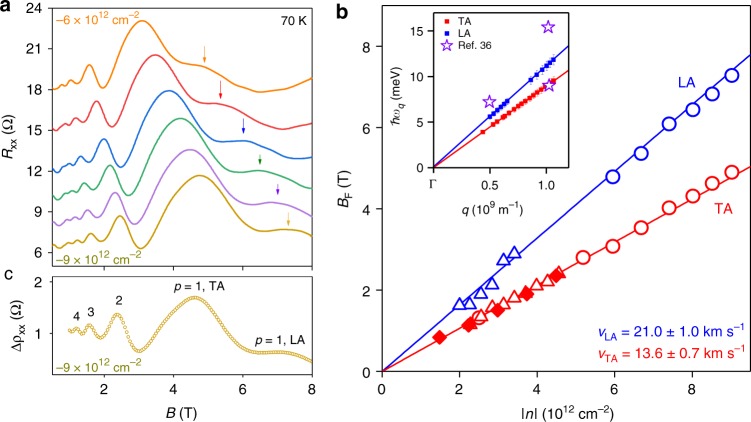
Phonon spectroscopy in graphene by measurement of magnetophonon oscillations. **a** Longitudinal resistance *R*_*xx*_ as a function of *B* measured for several high *n* of holes in our wide (*W* = 13.8 μm) dual-gated graphene Hall bar. The curves have been off-set vertically for clarity. **b** Red symbols plot the fundamental frequency *B*_F_ ≡ *pB*_p_ of magnetophonon oscillations as a function of absolute *n* for three different devices; open circles correspond to the dual-gated device which allowed high doping. The blue symbols mark the positions *B*_*p*=1_ of the broad peak (indicated by coloured arrows in **a**) which appears clearly at high *n*. The red and blue solid lines represents Eq. (1) with *v*_s_/*v*_F_ = 0.0128 and 0.0198 respectively. Knowing *v*_F_ (Supplementary Note 5) we extract the TA (*v*_TA_) and LA (*v*_LA_) phonon velocities. Inset: Data in main panel transformed to phonon dispersion curves. Coloured squares−experimental data points (error bars reflect the error in the experimentally extracted *v*_F_), solid lines plot the equation *ħω*_*q*_ = *ħv*_s_*q* (same *v*_s_ as in the main panel), purple stars—data taken from ref. ^36^. **c** Calculation of the oscillatory part of the resistivity Δ*ρ*_*xx*_(Ω) using the Kubo formula (see Supplementary Note 6 for details)

Equation (1) is generic for linearly dispersed phonons in graphene. This motivated us to search for MPR arising from longitudinal acoustic (LA) phonons, which should occur at higher *B* due to their significantly higher *v*_s_^34,35^. Careful inspection of the data in Figs. 1 and 2 shows that the *p* *=* 1 resonance for TA phonons is followed by a weak shoulder-like feature at higher *B*. We therefore studied a dual-gated graphene device (Supplementary Fig. 1) that permitted measurements at higher *n* ∼ −1 × 10^13^ cm^−2^ which, according to Eq. (1), should better separate this feature from the TA resonances. Figure 4a plots *R*_*xx*_ (*B*) for this device for several *n*. Measurements at these high *n* reveals that the shoulder-like feature develops into a well-defined peak (indicated by coloured arrows). Its position (*B*_*p*=1_) is accurately described by Eq. (1) with a constant *v*_s_/*v*_F_ = 0.0198. Using the experimentally extracted value of *v*_F_, we obtained *v*_s_ = 21.0 + 1.0 km s^−1^. This value is indeed close to that calculated for LA phonons in graphene^31,34,35^, and hence we attribute this feature to inter-LL scattering by LA phonons.

Further validation of our model is presented in Fig. 4b, which plots the magnetophonon oscillation frequency for TA phonons (*B*_F_ ≡ *pB*_*p*_) as a function of *n* for several different devices (red symbols). It shows that a linear dependence (red line) fits the data to Eq. (1) for all our measured devices over a range of *n* spanning an order of magnitude. The weaker LA resonance was also found to occur at the same *B*_*p*=1_ = *B*_F_ in different devices (blue symbols). Furthermore, the data in Fig. 4b can be transformed directly into phonon dispersion curves (inset of Fig. 4b) by noting that *q* ≈ 2*k*_F_ = 2(*nπ*)^0.5^ and *ħω*_*q*_ ≈ (2e*B*_F_*v*_s_*v*_F_*ħ*)^0.5^. The extended tunability of the carrier density in our dual-gated devices allows measurement of phonon branches up to wave vectors >10^9^ m^−1^. Note that these dispersion plots are significantly more precise than those measured by X-ray scattering experiments in graphite^36^ (purple stars). Studies of magnetophonon oscillations thus enable an all-electrical measurement of the intrinsic phonon dispersion curves in gate-tuneable materials.

## Discussion

To understand why magnetophonon oscillations are absent in narrow samples, we first note that figure of eight trajectories (Fig. 1b) have a spatial extent ∼4*R*_c_, which can reach values of several microns for the high-order resonances (*p* > 3). If the sample is too narrow, so that 4*R*_c_ is comparable to *W*, the carrier trajectories are skewed by elastic scattering at the device edges. In this case, they propagate along the edges of the device in skipping orbits^2^, effectively short-circuiting the resistive behaviour of the bulk caused by MPR_._ However, if *W* > 4*R*_c_, both MPR and skipping orbits contribute to *R*_*xx*_. We can estimate the width of the device required to observe MPR by comparing the relative contributions of these two processes. Carriers that diffuse in MPR-induced figure of eight trajectories move a distance 2*R*_c_ in a characteristic time, *τ*_e–ph_ = *L*_e–ph_/*v*_F_ with a drift velocity *v*_MPR_ = 2*R*_c_/*τ*_eph_. This is significantly slower than skipping orbits which can have speeds approaching *v*_F_. On the other hand, skipping orbits occupy only a width ∼*R*_c_ at each edge, whereas MPR occurs approximately over the full width, *W*, of the bulk. By comparing these two contributions, we deduce that MPR dominates when *Wv*_MPR_ ≳ 2*R*_c_*v*_F_. This corresponds to the condition *W* ≳ *L*_e–ph_, in good agreement with the measured data in Fig. 1d, f.

Our measurements provide an important insight into the intrinsic electron–phonon interaction in graphene: namely, the dominance of carrier scattering by low-energy TA phonons. This is in agreement with several theoretical works^35,37,38^ and contrasts with a widely held view that deformation potential scattering by LA phonons prevails over TA phonons^39^. To investigate this point further, we calculated magnetoresistance using the Kubo formula^40^ (Supplementary Note 6). A typical calculation is shown in Fig. 4c, which plots the contribution (Δ*ρ*_*xx*_) of MPR for TA and LA phonon velocities of *v*_s_ = 13.6 and 21.4 km s^−1^, respectively^35^, and the Fermi velocity^41^ *v*_F_ = 1 × 10^6^ m s^−1^. It accurately describes the oscillatory form of the measured data. Such good agreement is only possible when our calculations include the effect of carrier screening^35,38,42,43^ which significantly reduces the electron–LA phonon deformation potential coupling. Without screening, LA phonons would dominate the observed MPR (Supplementary Fig. 5). Our results therefore highlight the importance of carrier screening on electron–phonon interactions and thus helps resolve a long-standing discussion of the relative importance of LA^39,44^ and TA^37,38,43^ phonon scattering in graphene.

To conclude, we report the observation of pronounced magnetophonon oscillations in graphene, where the Dirac spectrum strongly modifies the resonant condition compared to previously studied electronic systems. Other two-dimensional crystals can also be expected to exhibit this phenomenon. The oscillations enable the study of low-energy acoustic phonon modes that are generally inaccessible by Raman spectroscopy^45,46^. Our measurements combined with the Kubo calculations provide strong evidence that TA phonons limit temperature-dependent mobility in graphene^35,37,38^. Most importantly, graphene's transport properties are shown to strongly depend on device size, even for conducting channels as wide as several microns. This should motivate further experiments on graphene and related two-dimensional materials in a macroscopic regime beyond the scope of previous mesoscopic devices.

## Methods

### Quantum transport measurements

For measuring resistance in our graphene devices, we used standard low-frequency AC measurement techniques with a lock-in amplifier at 10–30 Hz. The measurements of *R*_*xx*_(*Ω*) = *V*_*xx*_/*I*_*xx*_ are obtained by driving a small AC excitation current (*I*_*xx*_ = 0.1–1 μA) down the length of the Hall bar while simultaneously measuring the four probe voltage drop (*V*_*xx*_) between two side contacts located on the edge of the Hall bar devices (Fig. 1c). We tune the Fermi level in our graphene devices by applying a DC voltage between the silicon substrate and the graphene channel, where the SiO_2_ and bottom hexagonal boron nitride encapsulation layer serve as the dielectric (see Supplementary Note 1 for details on device fabrication). In our top gated device (see Supplementary Fig. 1), we simultaneously apply a potential to the metal top gate which allowed us to reach higher doping levels (see Fig. 4). All measurements were performed inside a variable temperature inset of a wet helium-4 flow cryostat that allowed us to carry out temperature-dependent magnetotransport measurements using a cold superconducting magnet.

## Supplementary information

Supplementary Information

## Data Availability

The data that support plots within this paper and other findings of this study are available from the corresponding author upon reasonable request.

## References

[1] Ando T, Fowler AB, Stern F (1982). Electronic properties of two-dimensional systems. Rev. Mod. Phys..

[2] Beenakker, C. W. J. & van Houten, H. in *Semiconductor Heterostructures and Nanostructures* (eds. Ehrenreich, H. & Turnbull, D. B. T.-S. S. P.) Vol. 44, 1–228 (Academic Press, California, 1991). London NW1 7DX (UK edition).

[3] Novoselov KS (2007). Room-temperature quantum Hall effect in graphene. Science.

[4] Taychatanapat T, Watanabe K, Taniguchi T, Jarillo-Herrero P (2013). Electrically tunable transverse magnetic focusing in graphene. Nat. Phys..

[5] Hunt B (2013). Massive Dirac fermions and Hofstadter butterfly in a van der Waals heterostructure. Science.

[6] Ponomarenko LA (2013). Cloning of Dirac fermions in graphene superlattices. Nature.

[7] Dean CR (2013). Hofstadter’s butterfly and the fractal quantum Hall effect in moiré superlattices. Nature.

[8] Li JIA (2017). Even-denominator fractional quantum Hall states in bilayer graphene. Science.

[9] Young AF, Kim P (2009). Quantum interference and Klein tunnelling in graphene heterojunctions. Nat. Phys..

[10] Levy N (2010). Strain-induced pseudo–magnetic fields greater than 300 Tesla in graphene nanobubbles. Science.

[11] Wang Y (2013). Observing atomic collapse resonances in artificial nuclei on graphene. Science.

[12] Krishna Kumar R (2017). High-temperature quantum oscillations caused by recurring Bloch states in graphene superlattices. Science.

[13] Dean CR (2010). Boron nitride substrates for high-quality graphene electronics. Nat. Nano.

[14] Mayorov AS (2011). Micrometer-scale ballistic transport in encapsulated graphene at room temperature. Nano Lett..

[15] Zibrov AA (2017). Tunable interacting composite fermion phases in a half-filled bilayer-graphene Landau level. Nature.

[16] Firsov YA, Gurevich VL, Parfeniev RV, Shalyt SS (1964). Investigation of a new type of oscillations in the magnetoresistance. Phys. Rev. Lett..

[17] Mashovets DV, Parfen’ev RV, Shalyt SS (1964). New data on the magnetophonon oscillation of the longitudinal magnetoresistance of N-TyPE InSb. J. Exp. Theor. Phys..

[18] Wood RA, Stradling RA (1968). The magnetophonon effect in III-V semiconducting compounds. J. Phys. C Solid State Phys..

[19] Nicholas RJ (1985). The magnetophonon effect. Prog. Quantum Electron..

[20] Greenaway MT (2015). Resonant tunnelling between the chiral Landau states of twisted graphene lattices. Nat. Phys..

[21] Gurevich VL, Firsov YA (1961). On the theory of the electrical conductivity of semiconductors in a magnetic field. J. Exp. Theor. Phys..

[22] Eaves L (1975). Fourier analysis of magnetophonon and two-dimensional Shubnikov-de Haas magnetoresistance structure. J. Phys. C Solid State Phys..

[23] Tsui DC, Englert T, Cho AY, Gossard AC (1980). Observation of magnetophonon resonances in a two-dimensional electronic system. Phys. Rev. Lett..

[24] Zudov MA (2001). New class of magnetoresistance oscillations: interaction of a two-dimensional electron gas with leaky interface phonons. Phys. Rev. Lett..

[25] Hatke AT, Zudov MA, Pfeiffer LN, West KW (2009). Phonon-induced resistance oscillations in 2D systems with a very high electron mobility. Phys. Rev. Lett..

[26] Morozov SV (2008). Giant intrinsic carrier mobilities in graphene and its bilayer. Phys. Rev. Lett..

[27] Wang L (2013). One-dimensional electrical contact to a two-dimensional material. Science.

[28] Barker JR (1972). The oscillatory structure of the magnetophonon effect. I. Transverse configuration. J. Phys. C Solid State Phys..

[29] Yu GL (2013). Interaction phenomena in graphene seen through quantum capacitance. Proc. Natl. Acad. Sci. USA.

[30] Elias DC (2011). Dirac cones reshaped by interaction effects in suspended graphene. Nat. Phys..

[31] Perebeinos V, Tersoff J (2009). Valence force model for phonons in graphene and carbon nanotubes. Phys. Rev. B.

[32] Falkovsky LA (2008). Symmetry constraints on phonon dispersion in graphene. Phys. Lett. A.

[33] Lindsay L, Broido DA (2010). Optimized Tersoff and Brenner empirical potential parameters for lattice dynamics and phonon thermal transport in carbon nanotubes and graphene. Phys. Rev. B.

[34] Karssemeijer LJ, Fasolino A (2011). Phonons of graphene and graphitic materials derived from the empirical potential LCBOPII. Surf. Sci..

[35] Sohier T (2014). Phonon-limited resistivity of graphene by first-principles calculations: electron-phonon interactions, strain-induced gauge field, and Boltzmann equation. Phys. Rev. B.

[36] Mohr M (2007). Phonon dispersion of graphite by inelastic x-ray scattering. Phys. Rev. B.

[37] Kaasbjerg K, Thygesen KS, Jacobsen KW (2012). Unraveling the acoustic electron-phonon interaction in graphene. Phys. Rev. B.

[38] Park C-H (2014). Electron–phonon interactions and the intrinsic electrical resistivity of graphene. Nano Lett..

[39] Hwang EH, Das Sarma S (2008). Acoustic phonon scattering limited carrier mobility in two-dimensional extrinsic graphene. Phys. Rev. B.

[40] Kubo, R., Miyake, S. J. & Hashitsume, N. in *Solid State Phyics* (eds. Seitz, F. & Turnball, D. (Academic, New York, NY, 1965).

[41] Castro Neto AH, Guinea F, Peres NMR, Novoselov KS, Geim AK (2009). The electronic properties of graphene. Rev. Mod. Phys..

[42] Ni GX (2018). Fundamental limits to graphene plasmonics. Nature.

[43] von Oppen F, Guinea F, Mariani E (2009). Synthetic electric fields and phonon damping in carbon nanotubes and graphene. Phys. Rev. B.

[44] Suzuura H, Ando T (2002). Phonons and electron-phonon scattering in carbon nanotubes. Phys. Rev. B.

[45] Kossacki P (2012). Circular dichroism of magnetophonon resonance in doped graphene. Phys. Rev. B.

[46] Kim Y (2013). Measurement of filling-factor-dependent magnetophonon resonances in graphene using raman spectroscopy. Phys. Rev. Lett..

