# Supplementary material for: Strong magnetophonon oscillations in extra-large graphene
Source: Nat Commun. 2019 Jul 26;10:3334. doi: 10.1038/s41467-019-11379-3 (PMC6659705; doi:10.1038/s41467-019-11379-3)
Supplement: Supplementary file 1 — Supplementary Information [file 41467_2019_11379_MOESM1_ESM.pdf]

Supplementary Information

Strong magnetophonon oscillations in extra-large graphene

P. Kumaravadivel[1,2], M. T. Greenaway[3,4], D. Perello[1,2], A. Berdyugin[1], J. Birkbeck[1,2], J. Wengraf[1,5], S. Liu[6], J. H. Edgar[6], A. K. Geim[1,2], L. Eaves[1,4], R. Krishna Kumar[1]

[1]School of Physics & Astronomy, University of Manchester, Manchester M13 9PL, UK

[2]National Graphene Institute, University of Manchester, Manchester M13 9PL, UK

[3]Department of Physics, Loughborough University, Loughborough, UK

[4]School of Physics & Astronomy, University of Nottingham, Nottingham, UK

[5]Department of Physics, University of Lancaster, Lancaster LA1 4YW, UK

[6]Department of Chemical Engineering, Kansas State University, Manhattan, KS 66506, USA

Supplementary Note 1: Device Fabrication

The hexagonal boron nitride (hBN) encapsulated graphene heterostructures were assembled using a peel and stack deterministic dry transfer method. Graphene and hBN flakes were first exfoliated onto O_2/Ar plasma cleaned SiO_2/Si substrates. The appropriate flakes were then identified by long exposure dark field optical imaging. For the hBN encapsulation layers, we used flakes that were 25 – 100 nm thick. The selected flakes were then assembled using a polypropyl carbonate (PPC) coated Polydimethylsiloxane (PDMS) stamp placed on a glass slide attached to a high precision XYZ micromanipulator[1]. First, the top hBN encapsulation layer was picked up using the PPC/PDMS stamp. This was done at a fixed substrate temperature ~ 50^0 C. The hBN flake attached to the stamp was then used to pick up the graphene flake. During this process, the substrate temperature was fixed at 65° C whilst smoothly contacting hBN to graphene by fine Z adjustments of the micromanipulator. Once fully contacted at 50° C the resultant hBN/graphene stack was peeled off from the substrate onto the PPC/PDMS stamp. The stack was then placed down on the bottom hBN flake (substrate temperature at 65°) to fully encapsulate the graphene layer.

Once the heterostructure was prepared, we performed standard electron beam lithography techniques to create the Hall bar geometry. First, we patterned a polymethyl methacrylate (PMMA) mask on the stack to define contact regions leading up to the device channel. The regions unprotected by the mask were etched away using $CHF_3 + O_2$ reactive ion etching (RIE), forming narrow trenches. Metal contacts (5 nm Cr/70 nm Au) were then evaporated into the trenches which form high-quality contacts to the graphene edge[2,3]. Next, the same lithography and RIE etching procedures were used to pattern the Hall bar mesa. For one of our devices, we also patterned a metallic top gate above the heterostructure (Supplementary Figure 1) which allowed us to achieve higher doping levels.

Supplementary Note 2: Magnetic Focussing in wide graphene Hall bars

At low temperature, the charge carriers in our graphene/hBN devices propagate ballistically for several micrometres before scattering. For certain measurement geometries, the 4-probe resistance is governed by direct transmission of charge carriers between current and voltage probes. An example is illustrated in Supplementary Figure 2a which describes the measurement scheme for a transverse magnetic focussing experiment. In the presence of a perpendicular magnetic field (B), ballistic carriers injected from the current source follow curved trajectories and the measured 4-probe resistance (R_{TMF}) exhibits maxima for particular values of B when the carriers are focussed directly into the collector voltage probe (coloured arrows in Supplementary Figure 2a). This occurs when the cyclotron radius ($R_c \sim \hbar k_F/eB$) becomes commensurate with the distance (L) between the current injector and voltage collector. The resonance condition is given by

$$B = \frac{2\hbar k_{\mathrm{F}} s}{eL} \qquad \text{Supplementary Equation (1)}$$

where k_{F} is the Fermi wave-number and (s-1) is an integer number that describes the number of reflections at the device edge. For example, s = 2 describes the trajectories traced by the red arrows in Supplementary Figure 2a. Supplementary Figure 2b shows R_{TMF} (B) at fixed n measured in the geometry illustrated in Supplementary Figure 2a. For negative values of B, we find a set of maxima that are equally spaced with a period $\Delta B = 2\hbar k_{\mathrm{F}}/eL$. No resonances are observed for positive B because

the Lorentz force acts in the opposite direction and bends trajectories away from the voltage collector. Supplementary Figure 2c plots maps of R_{TMF} (B,n) for different hole doping. We find the resonances are shifted to higher B for higher n, in agreement with Supplementary Equation 1 and the fact that the cyclotron radius is larger for higher n. Notably, all the resonances can be described by Supplementary Equation 1 with L = 7.4 μm (dashed lines in Supplementary Figure 2c). This value corresponds to the distance between the current injector and voltage collector (labelled in Supplementary Figure 2c) and validates the dependence of the oscillatory features given by Supplementary Equation 1.

Magnetic focussing resonances can also appear in a standard longitudinal resistance measurement (R_{xx}) if the voltage probes are located too close to the current contacts (see main text). This occurs if they are closer than the width of the channel and is typically the case in our wider samples (Supplementary Figure 2d) because of the limited size of exfoliated flakes and clean areas available for patterning devices[4,5]. Supplementary Figure 2e plots R_{xx} (B) for a fixed n and T in non-quantizing B fields. We find a set of resonances that are periodic in B and occur at higher B for higher n (Supplementary Figure 2f). This behaviour resembles that of magnetic focussing although these curves are distinct from a typical measurement (Supplementary Figures 2a-c). First, the current injectors are rather wide (15 μm). Second, the oscillations are slightly phase shifted; whereas resonances are equally spaced with a period ΔB, the first (labelled 1 in Supplementary Figure 2e) occurs at a value approximately $\Delta B/2$. To understand the origin of those resonances, we used Supplementary Equation 1 to extract L from the oscillation period $\Delta B = 2\hbar k_F/eL$. We carried out this analysis for several different doping levels (Supplementary Figure 2f) and found approximately the same $L \approx 5.6$ μm. This corresponds roughly to the distance between current and voltage probes and suggests that the dominant contribution to the measurement originates from magnetic focussing where carriers are injected from the corners of the device (yellow arrows in Supplementary Figure 2d). Further work is required to understand the details of these magnetic focussing resonances.

Supplementary Note 3: Quasi-ballistic device channels

Figure. 1f of the main text shows that magnetophonon oscillations in graphene appear only in samples in which the channel has a sufficiently large width (W). We argue that the size is the important variable because all our devices exhibit similar electronic quality in the bulk; their conducting channels are relatively free from defects/impurities so that low-temperature mobility is limited only by scattering at the edges[6]. To prove this, we performed magnetic focussing experiments (described in Supplementary Section 2) on all our devices. The observation of magnetic focussing resonances requires carriers that propagate ballistically without scattering, thus proving there are no scattering centres along the path between the current injector and voltage collector. Here we present data taken from transverse magnetic focussing experiments performed in our narrowest (W = 1.5 μm) and widest (W = 15 μm) samples. The measurement geometries for each device are sketched in the Supplementary Figures 3a-b. Supplementary Figure 3c plots R_{TMF} (n,B) measured in the narrowest device in which injector and collector probes are separated by L = 1.5 μm. We find pronounced magnetic focussing resonances up to the fourth order (s = 4). We note that similar resonances also appear between any pairs of contacts located in different regions of the device, thus providing further confirmation of the quality and ballistic nature of our channels. Even in our widest samples

(Supplementary Figure 3b) we detect ballistic electrons focused at voltage probes 20 μm away from current injector (Supplementary Figure 3d).

Supplementary Note 4: Semiclassical model of magnetophonon resonance in monolayer graphene

The magnetoresistivity of the device is given by the approximation

$$\rho_{yy} \approx \frac{\sigma_{xx}}{\sigma_{xy}^2} \approx \rho_{xx}, \qquad \text{Supplementary Equation (2)}$$

since the Hall component of the conductance tensor, $\sigma_{xy} >> \sigma_{xx}$ for these experimental conditions. The longitudinal conductance tensor component, σ_{xx}, is determined by the rate of drift of a carrier's cyclotron orbit centre. This process is illustrated semiclassically in Fig. 1b of the main text which shows the shift caused by an inelastic scattering-induced figure–of-eight transition in *k*-space. The energy absorbed (or emitted) by the carrier undergoing an inter-Landau level (LL) transition is given by the energy difference between its initial (*N*) and final ($N \pm p$) states, where *N* is the LL index and *p* is a positive integer. The quantised energy spectrum of monolayer graphene is given by

$$E_N = \mathrm{sgn}(N)\sqrt{2|N|}\,\frac{\hbar v_{\mathrm{F}}}{l_B}, \qquad \text{Supplementary Equation (3)}$$

where v_F is the Fermi velocity of graphene and $l_B = \sqrt{\hbar/eB}$ is the quantum magnetic length. For a figure-of-8 transition, an electron in a LL with index N and orbit radius in *k*-space given by $\kappa_{\mathrm{c}} = \sqrt{2N}/l_B$ makes an inelastic transition to a level with index $N' = N \pm p$ by absorbing or emitting a phonon with wave vector, q, so that its final radius $\kappa_{\mathrm{c}}' = q - \kappa_{\mathrm{c}}$. In real space, the corresponding classical orbits have radii $R_{\mathrm{c}} = l_B^2 \kappa_{\mathrm{c}}$. The wavevector, q induces a shift in real space of the orbit centre $\Delta X = l_B^2 q$ and hence provides a contribution to the current. An excellent fit to the measured period of the magnetophonon oscillations is obtained by considering scattering by linearly dispersed acoustic phonons with energy $\hbar\omega_q = \hbar v_{\mathrm{s}} q = \hbar v_{\mathrm{s}}(\kappa_{\mathrm{c}}' + \kappa_{\mathrm{c}})$, where v_{s} refers to velocity of either longitudinal acoustic (LA) or transverse acoustic (TA) phonons. The resonant conditions for absorption or emission processes are given by

$$\hbar\omega_q = \frac{\hbar v_{\mathrm{s}}}{l_B}\left(\sqrt{2(N \pm p)} + \sqrt{2N}\right) = \pm\frac{\hbar v_{\mathrm{F}}}{l_B}\left(\sqrt{2(N \pm p)} - \sqrt{2N}\right), \qquad \text{Supplementary Equation (4)}$$

where *p* is the change in LL index. This can be expressed in the following form

$$N = \frac{p v_{\mathrm{s}}}{4 v_{\mathrm{F}}}\left(\frac{v_{\mathrm{F}}}{v_{\mathrm{s}}} - 1\right)^2 \approx \frac{p v_{\mathrm{F}}}{4 v_{\mathrm{s}}}. \qquad \text{Supplementary Equation (5)}$$

The approximation holds since $v_{\mathrm{s}} \ll v_{\mathrm{F}}$. Only electrons within the energy range $\sim(E_{\mathrm{F}} \pm 2k_{\mathrm{B}}T)$, can scatter between filled and empty states by emitting or absorbing a phonon. The Fermi energy $E_{\mathrm{F}} \gg 2k_{\mathrm{B}}T$ over the temperature range of our measurements. The LL index, *N,* of the carriers undergoing MPR transitions around the Fermi energy is proportional to the carrier density, $n = 4eBN/h$, where the factor 4 corresponds to the two-fold valley and spin degeneracies. Combining this relation with (S5) we obtain the magnetic field position B_p of the pth resonant peak:

$$B_p = \frac{nhv_{\mathrm{F}}}{pev_{\mathrm{s}}}\left(\frac{v_{\mathrm{F}}}{v_{\mathrm{s}}} - 1\right)^{-2} \approx \frac{nhv_{\mathrm{s}}}{pe\ {}_{\mathrm{F}}}. \qquad \text{Supplementary Equation (6)}$$

Therefore, the magnetophonon resonance oscillations are periodic in $1/B$ with a frequency $B_F = pB_p$, as discussed in the main text.

Supplementary Note 5: Fermi velocity of charge carriers in graphene encapsulated with hexagonal boron nitride

Figure. 3 of the main text demonstrates that the position of maxima in the resistance caused by magnetophonon resonance can be described by Supplementary Equation 6 with the only fitting parameter being the ratio of the phonon speed to the Fermi velocity in graphene v_s/v_F. Knowing the Fermi velocity of graphene we are able to measure the speed of the phonons responsible for the observed effect. It can be obtained by studying temperature dependence of Shubnikov de Haas (SdH) oscillations[7] and its measurement allows us to extract v_s without any fitting parameters. Supplementary Figure S4a plots the temperature dependence of the magnetoresistivity $\rho_{xx}(B)$ for one of our graphene devices encapsulated with hBN. We observe pronounced 1/B-periodic SdH oscillations that are almost completely damped at 50 K (red curve in Supplementary Figure S4a). The inset of Supplementary Figure S4a plots the amplitude of one of the oscillations (indicated by black arrow in the main panel) as a function of T. It shows that the data can be fitted precisely by the Lifshitz-Kosevich formula (solid red line) which allows us to extract the effective mass m^*. We repeated such measurement and analysis of SdH oscillations in different devices and for a range of n between 1 and 4 x 10^{12} cm^{-2}. These density dependent measurements reveal a linear dependence of m^* as function of the Fermi wavevector $k_F = (n\pi)^{0.5}$ (Supplementary Figure S4b), as previously observed for graphene on dielectric substrates[7,8]. Fitting the experimental data with the standard equation for $m^* = \hbar k_F/v_F$ in graphene[9] allows us to extract $v_F = 1.06 \pm 0.05 \times 10^6$ ms^{-1}. This value is in good agreement with previous measurements[7,8] and that which is typically expected for graphene[9] at these relatively high n where velocity renormalisation due to e-e interactions[10] is negligible.

Supplementary Note 6: Summary of quantum transport calculations

We use the Kubo approach[11] to determine the linear response of the oscillatory longitudinal magnetoresistivity, ρ_{xx}, of monolayer graphene due to the resonant absorption and emission of LA and TA acoustic phonons by the charge carriers in a magnetic field, $\mathbf{B} = (0,0,-B)$, applied perpendicular to the graphene sheet. The model corresponds to ohmic conditions with the carriers in thermal equilibrium with the lattice vibrations. The electronic spectrum becomes quantised into a series of unevenly spaced Landau levels (LLs) with index N given by Supplementary Equation 3. It is convenient to use the Landau gauge where $\mathbf{A} = (0, -Bx, 0)$. The carrier wave function in the K^+ valley and the conduction band is then given by the pseudospinor

$$\psi_N^{K^+} = \frac{1}{\sqrt{2}} \begin{pmatrix} \phi_{|N|}(x-X) \\ -\mathrm{sgn}(N)\mathrm{i}\phi_{|N|-1}(x-X) \end{pmatrix}, \qquad \text{Supplementary Equation (7)}$$

where ϕ are simple harmonic oscillator states along x and plane waves along y given by

$$\phi_N(x) = A_N H_N\left(\frac{x}{l_B}\right) \exp\left(\frac{x^2}{2l_B^2}\right) \exp(\mathrm{i}k_y y), \qquad \text{Supplementary Equation (8)}$$

Here $A_N = 1/\sqrt{L_y l_B 2^N N! \sqrt{\pi}}$ is a normalisation constant and H_N are the Hermite polynomials[12,9,13]. A similar relation applies for the valence band and the K^- valley. In the Kubo approach, the contribution of the TA and LA acoustic phonon scattering to the magnetoconductance σ_{xx} is given by

$$\Delta\sigma_{xx}^{\mathrm{t,l}} = \frac{g_{\mathrm{v}} g_{\mathrm{s}} \pi e^2}{S^2 k_{\mathrm{B}} T \hbar} \sum_{\mathbf{q}} \left(l_B^2 q_y\right)^2 |C\,(q)|^2 N_q \left(N_q + 1\right) \sum_{N,N'} \sum_{k_y, k_y'} \left[f\left(E_N - \hbar\omega_q^{\mathrm{t,l}}\right) - f(E_N)\right] \delta\left(E_N - \hbar\omega_q^{\mathrm{t,l}} - E_{N'}\right) \left|I_{N,N'}^{\mathrm{t,l}}(k_y, k_y', \mathbf{q})\right|^2 . \quad \text{Supplementary Equation (9)}$$

Here the subscripts/superscripts t and l refer to contributions corresponding to the TA or LA phonons respectively, $g_{\mathrm{v}} = 2$ and $g_{\mathrm{s}} = 2$ are the valley and spin degeneracies, $S = L_x L_y$ is the area of the device, k_{B} is the Boltzmann constant, T is the lattice temperature, $\left|C_{\mathrm{t,l}}(q)\right|^2 = \hbar q/(2\rho v_{\mathrm{t,l}})$ are the Fourier components of the scattering potential, $\rho = 7.6 \times 10^{-8}$ g cm^{-2} is the mass density of graphene, $N_q = (\exp(\hbar\omega_q^{\mathrm{t,l}}/k_{\mathrm{B}}T) - 1)^{-1}$ is the Bose-Einstein distribution function for the phonons and $f(E) = (\exp((E-\mu)/k_{\mathrm{B}}T) + 1)^{-1}$ is the Fermi-Dirac distribution of the carriers with chemical potential, μ. The matrix elements $I_{N,N'}^{\mathrm{t,l}}(\mathbf{q})$, are given by

$$I_{N,N'}^{\mathrm{t,l}}\left(k_y, k_y', \mathbf{q}\right) = \int dS \psi_{k_y', N'}^{*} V_{\mathbf{q}}^{\mathrm{t,l}} \psi_{k_y, N}. \quad \text{Supplementary Equation (10)}$$

$V_{\mathbf{q}}^{\mathrm{t,l}}$ describes the charge-carrier phonon coupling for the TA and LA phonons respectively[14,15,16,17] and has the form

$$V_{\mathbf{q}}^{\mathrm{t}} = e^{\mathrm{i}\mathbf{q}\cdot\mathbf{r}} \begin{pmatrix} 0 & -g_{\mathrm{g}} \mathrm{e}^{i2\varphi} \\ g_{\mathrm{g}} \mathrm{e}^{-i2\varphi} & 0 \end{pmatrix} \quad \text{Supplementary Equation (11)}$$

and

$$V_{\mathbf{q}}^{\mathrm{l}} = \mathrm{i} e^{\mathrm{i}\mathbf{q}\cdot\mathbf{r}} \begin{pmatrix} g_{\mathrm{d}}(q) & g_{\mathrm{g}} \mathrm{e}^{\mathrm{i}2\varphi} \\ g_{\mathrm{g}} \mathrm{e}^{-\mathrm{i}2\varphi} & g_{\mathrm{d}}(q) \end{pmatrix}, \quad \text{Supplementary Equation (12)}$$

where φ is the angle between the phonon wave-vector and the x axis, which in our model is defined to be along the zigzag edge of the graphene layer. Here, g_{g} and $g_{\mathrm{d}}(q)$ are the carrier-phonon coupling matrix elements corresponding to the gauge and deformation distortions of the graphene lattice. The off-diagonal matrix elements involving g_{g} arise from pure shear-like distortions of the lattice which give rise to a "synthetic" gauge field in the Dirac equation. This is unaffected by screening and has been estimated using density functional theory to have a value in the range g_{g} = 1.5 – 4.5 eV[14,15,16,17,18]. We obtain a good fit to the data with g_{g} = 4 eV. The on-diagonal terms correspond to strain-induced distortions of the unit cells that change their areas, resulting in local redistributions in the carrier density, *n,* which screen the deformation potential terms $g_{\mathrm{d}}(q)$. Therefore we write

$$g_{\mathrm{d}}(q) = \frac{\tilde{g}_{\mathrm{d}}}{\varepsilon(q)}, \quad \text{Supplementary Equation (13)}$$

where $\tilde{g}_{\mathrm{d}}$ is the unscreened "bare" deformation potential coefficient and $\varepsilon(q)$ is the phonon wave vector-dependent dielectric function. We use the Thomas-Fermi approximation for $\varepsilon(q)$ which gives

$$\varepsilon(q) = \varepsilon_{\mathrm{r}} \left(1 + \frac{q_{\mathrm{tf}}}{q}\right), \quad \text{Supplementary Equation (14)}$$

where $q_{\mathrm{tf}} = 4e^2\sqrt{n\pi}/(4\pi\hbar v_{\mathrm{F}}\varepsilon_{\mathrm{r}}\varepsilon_0)$ is the inverse Thomas-Fermi screening radius. This takes into account screening by the dielectric environment of the graphene layer, with the dielectric constant ε_{r} and by the free carriers in the graphene layer[18,19]. For free-standing graphene and ε_{r} = 1, $q_{\mathrm{tf}}\sim 8k_{\mathrm{F}}$. For the case of magnetophonon resonance at high LL index, $q \sim 2k_{\mathrm{F}}$ and $\varepsilon(q)\sim 5$ and thus the deformation potential is strongly suppressed[18]. In our experiments, the graphene layer is fully encapsulated by hBN and therefore we set ε_{r} = 3.5 so that $\varepsilon(q)\sim$ 7.5 and $g_{\mathrm{d}}(q)$ is reduced further. However, we find that the resistivity is not very sensitive to ε_{r} since the deformation potential is very effectively screened by the carriers in the graphene layer. We set $\tilde{g}_{\mathrm{d}}$ to be 25 eV. We find that in the range from $\tilde{g}_{\mathrm{d}} = 20 - 30$ eV the resistivity is not sensitive to this parameter due to the strong screening effect.

To model LL broadening, we replace the delta function in Supplementary Equation 9 by

$$\delta(E) \rightarrow \frac{1}{\Gamma\sqrt{2\pi}}\exp\left(-\frac{E^2}{2\Gamma^2}\right) \quad , \qquad \text{Supplementary Equation (15)}$$

where $E = E_N - E_{N'} - q\hbar v_{\mathrm{t,l}}$. We use a Gaussian function to aid convergence of our calculation at high LL indicies. In the case of short-range scattering, for example by charged impurities, the broadening of the LLs depends on the square root of magnetic field[12,20,21,22]. Therefore, we set the broadening parameter $\Gamma = \gamma\sqrt{B}$. We obtain a good fit to the data with γ = 0.5 meV $T^{-1/2}$ (see Fig. 4b of the main text).

In the Hall regime, the longitudinal magnetoresistivity is given by $\rho_{yy} = \sigma_{xx}/(\sigma_{xx}\sigma_{yy} + \sigma_{xy}^2)$, where the component $\sigma_{xy} = ne/B$. Since $\sigma_{xx} = \sigma_{yy} \ll \sigma_{xy}$ the oscillatory part of ρ_{xx} due to TA and LA phonon scattering is given by

$$\Delta\rho_{xx} = \left(\frac{B}{ne}\right)^2 \left(\sigma_{xx}^{\mathrm{l}} + \sigma_{xx}^{\mathrm{t}}\right) \qquad \text{Supplementary Equation (16)}$$

to a good approximation. Supplementary Figure 5 shows $\Delta\rho_{xx}$ calculated for three representative carrier densities, 6, 7.5 and 9 x 10^{12} cm^{-2}. We find that $\Delta\rho_{xx}(B)$ has a form and amplitude which agrees well with oscillations observed in the measurements shown in the main text. The magnetic field values of the position of the peaks correspond closely to the classical resonance condition for two touching cyclotron orbits, see Equation 1 of the main text and Supplementary Equation 5. The slight difference between the classical and quantum models arises from the form of overlap integral between the corresponding Landau-quantised wavefunctions. The peaks are periodic in 1/*B* with a frequency, B_F, that has a linear dependence on n (Supplementary Equation 6). The plot shows that the contribution from the LA phonons to the total resistivity is relatively small and appears only as the *p* = 1 peak in $\Delta\rho_{xx}$ (labelled by the blue arrow in Supplementary Figure 5). This is due to two main factors: first, the suppression of the deformation part of the electron-phonon coupling matrix due to free carrier screening (see Supplementary Equation 12); second, the energy of the LA phonon is larger than that of the TA phonon when the condition for magnetophonon resonance is satisfied. Therefore, at a given temperature, there is a lower population of LA phonons than TA phonons for carriers to absorb and similarly there are fewer carriers at high enough energy to emit an LA phonon compared to those that can emit a TA phonon. If the deformation part of the electron-phonon were not screened, $\rho_{xx}(B)$ would be dominated by the contribution from the LA phonons, highlighting the importance of the strong screening of LA phonon scattering in graphene.

Supplementary Figures

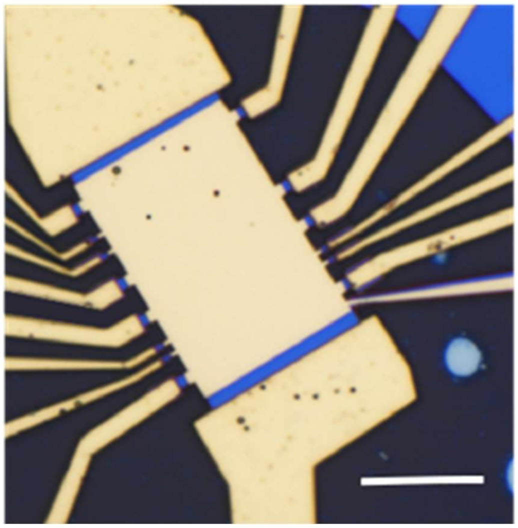

Supplementary Figure 1: Wide graphene Hall bars. Optical image of our wide top-gated device (W = 13.8 μm)**.** White scale bar is 10 μm.

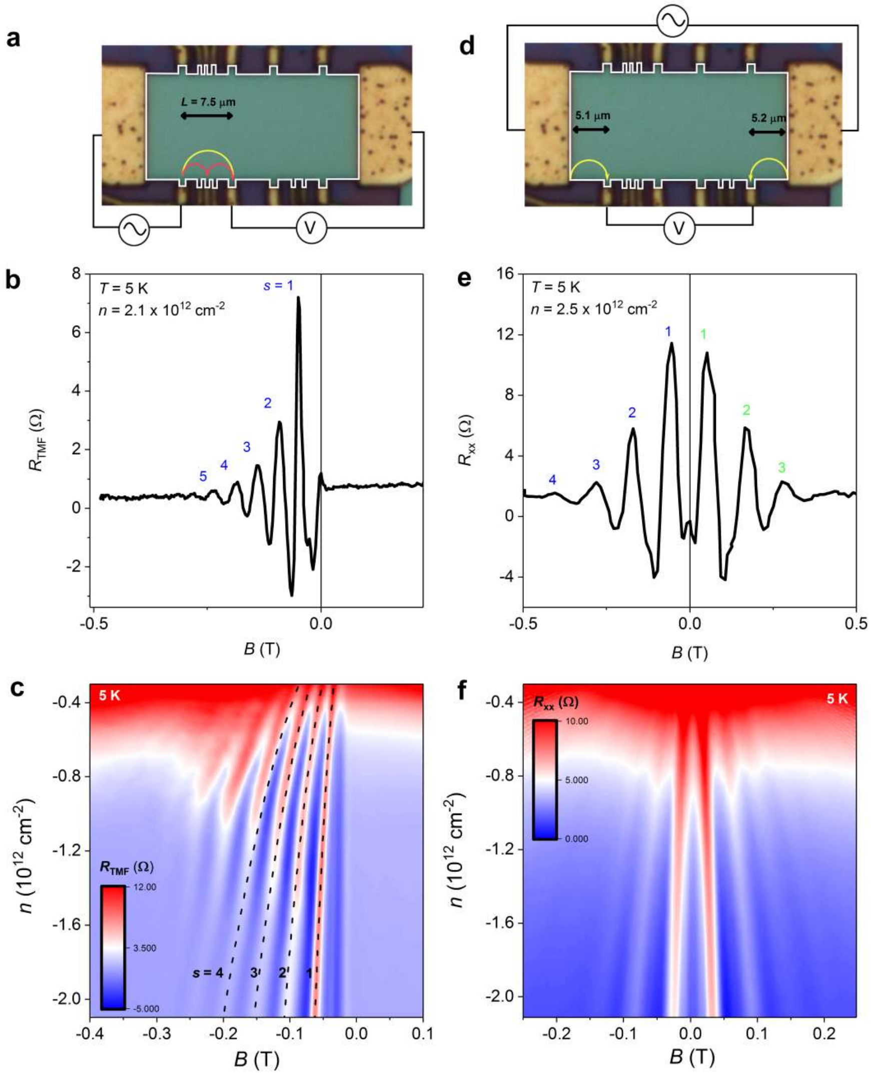

Supplementary Figure 2: Magnetic Focussing. a, Measurement scheme for a typical transverse magnetic focussing experiment performed in a graphene device with W = 15 um. Yellow and red lines trace trajectories of electrons under the resonance condition (1) for s = 1 and 2 respectively. **b**, R_{TMF} (B) for fixed n and T. **c**, Magnetic focussing maps, R_{TMF} (B, n), for the configuration specified in **a**. Dashed lines are fits to Supplementary Equation 1 with L = 7.4 μm and different s. **d** Measurement scheme for R_{xx} geometry; the yellow arrows depict trajectories of charge carriers in both negative and positive B-fields. **e,** R_{xx} (B) for a fixed n and T. **f**, maps R_{xx} (B, n).

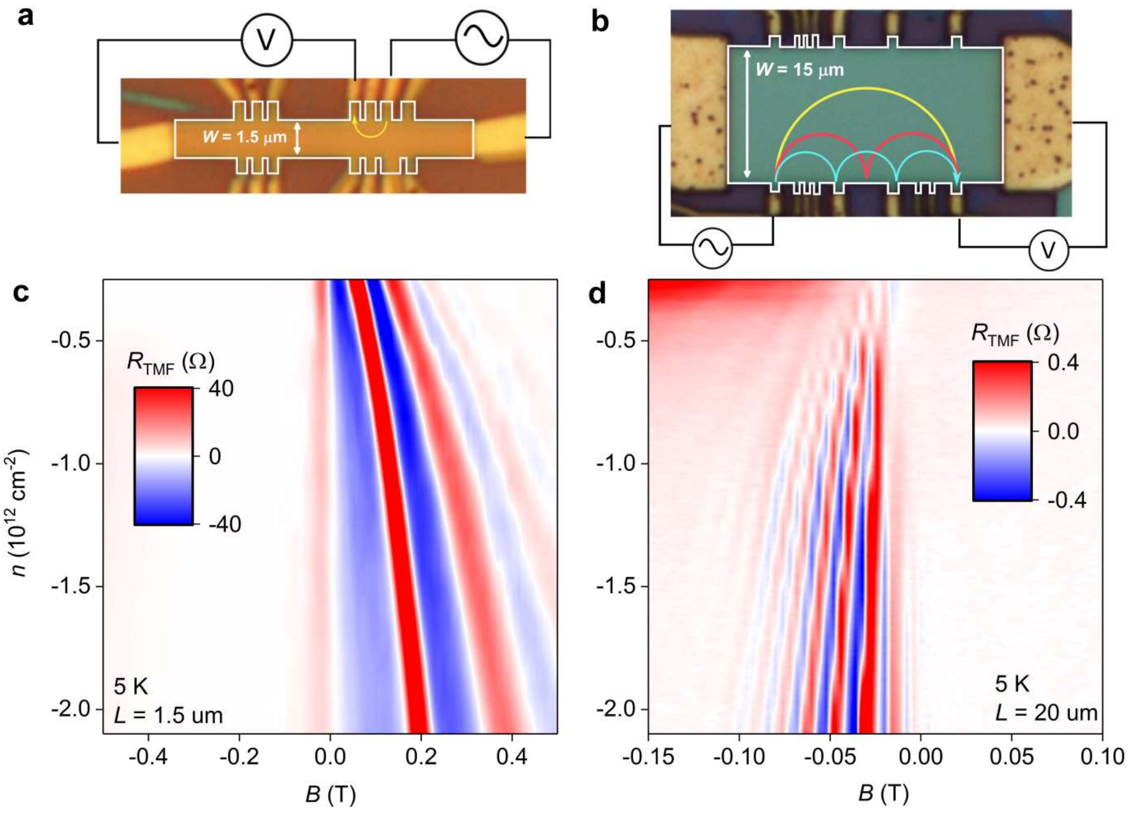

Supplementary Figure 3: Quasi-ballistic graphene devices. a,b, Measurement scheme for a magnetic focussing experiment performed in our narrowest (**a**) and widest (**b**) Hall bar devices (the mesa are contoured in white). The yellow, red and blue curved arrows indicate electron trajectories corresponding to resonances predicted by Supplementary Equation 1 with s = 1, 2 and 3 respectively. **c,d** Transverse magnetic focussing maps, R_{TMF} (n, B) measured at 5 K in the configurations shown in **a** and **b** respectively.

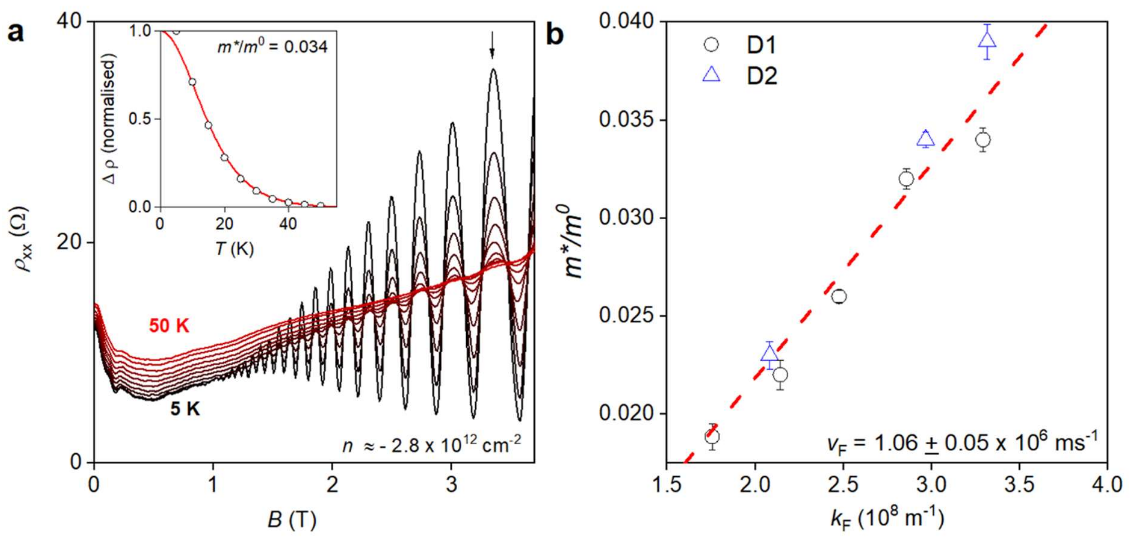

Supplementary Figure 4: Temperature dependence of Shubnikov de Hass oscillations. a, Magnetoresistance $\rho_{xx}(B)$ measured at a fixed hole density n = - 2.8 x 10^{12} cm^{-2} for different T in 5 K steps. We subtract the smooth background and analyse the amplitude of SdH oscillations (indicated by black arrow). Inset: T dependence of the normalised amplitude $\Delta\rho(T)$ for the peak that is indicated by the black arrow in **a**. Open circles are experimental data points and solid red line is the standard fitting with the Lifshitz-Kosevich formula. m^0 represents the free electron mass **b,** measurements of m^*/m^0 for different n of holes in two different graphene devices; the black circles represent data obtained in one of our widest devices W = 15 μm (Fig. 1c of the main text). The error bars represent the error in fitting the Lifshitz-Kosevich formula. Red dashed line is a linear fit to the equation $m^* = \hbar k_F/v_F$ with a constant v_F = 1.06 x 10^6 ms^{-1}.

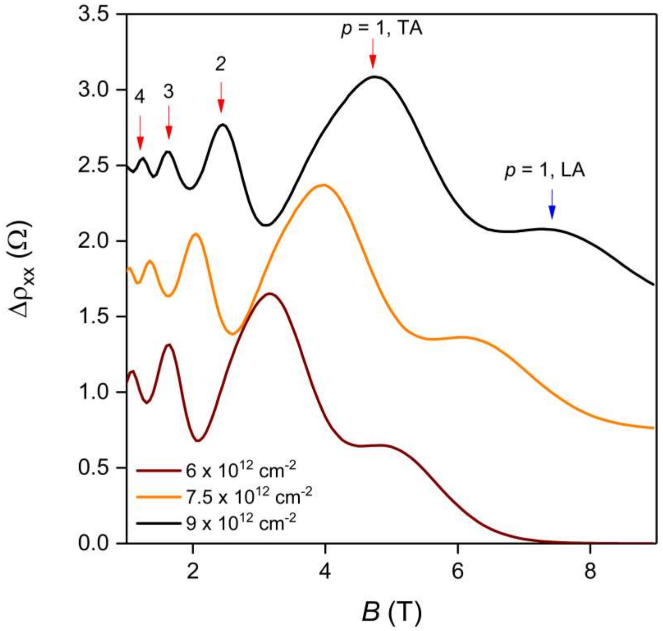

Supplementary Figure 5: Quantum transport calculations: Calculated $\Delta\rho_{xx}(B)$ for three different *n* and *T* = 70 K, using v_{t} = 13.6 kms^{-1} and v_{l} = 21.4 kms^{-1} see ref. 17 and the parameters specified in the text. The integers p correspond to resonant inter-LL scattering around the Fermi energy, with $p = |N - N'|$. The curves are offset for clarity.

Supplementary References

1. Frisenda, R. *et al.* Recent progress in the assembly of nanodevices and van der Waals heterostructures by deterministic placement of 2D materials. *Chem. Soc. Rev.* **47,** 53–68 (2018).

2. Wang, L. *et al.* One-Dimensional Electrical Contact to a Two-Dimensional Material. *Science.* **342,** 614–617 (2013).

3. Ben Shalom, M. *et al.* Quantum oscillations of the critical current and high-field superconducting proximity in ballistic graphene. *Nat. Phys.* **12,** 318-322 (2015).

4. Kretinin, A. V *et al.* Electronic Properties of Graphene Encapsulated with Different Two-Dimensional Atomic Crystals. *Nano Lett.* **14,** 3270–3276 (2014).

5. Haigh, S. J. *et al.* Cross-sectional imaging of individual layers and buried interfaces of graphene-based heterostructures and superlattices. *Nat. Mater.* **11,** 764-767 (2012).

6. Halbertal, D. *et al.* Imaging resonant dissipation from individual atomic defects in graphene. *Science***358,** 1303-1306 (2017).

7. Novoselov, K. S. *et al.* Two-dimensional gas of massless Dirac fermions in graphene. *Nature* **438,** 197–200 (2005).

8. Zhang, Y., Tan, Y.-W., Stormer, H. L. & Kim, P. Experimental observation of the quantum Hall effect and Berry's phase in graphene. *Nature* **438,** 201–204 (2005).

9. Castro Neto, A. H., Guinea, F., Peres, N. M. R., Novoselov, K. S. & Geim, A. K. The electronic properties of graphene. *Rev. Mod. Phys.* **81,** 109–162 (2009).

10. Elias, D. C. *et al.* Dirac cones reshaped by interaction effects in suspended graphene. *Nat Phys* **7,** 701–704 (2011).

11. Kubo, R., Miyake, S. J. & Hashitsume, N. :*in Solid State Phyics, ed. F Seitz and D. Turnball*. ((Academic, New York), 1965).

12. Shon, N. H. & Ando, T. Quantum Transport in Two-Dimensional Graphite System. *J. Phys. Soc. Japan* **67,** 2421–2429 (1998).

13. McCann, E. in (ed. Raza, H.) 237–275 (Springer Berlin Heidelberg, 2012). doi:10.1007/978-3-642-22984-8_8

14. Suzuura, H. & Ando, T. Phonons and electron-phonon scattering in carbon nanotubes. *Phys. Rev. B* **65,** 235412 (2002).

15. Mariani, E. & von Oppen, F. Temperature-dependent resistivity of suspended graphene. *Phys. Rev. B* **82,** 195403 (2010).

16. von Oppen, F., Guinea, F. & Mariani, E. Synthetic electric fields and phonon damping in carbon nanotubes and graphene. *Phys. Rev. B* **80,** 075420 (2009).

17. Sohier, T. *et al.* Phonon-limited resistivity of graphene by first-principles calculations: Electron-phonon interactions, strain-induced gauge field, and Boltzmann equation. *Phys. Rev. B* **90,** 125414 (2014).

18. Katsnelson, M. I. *Graphene: Carbon in Two Dimensions*. (Cambridge University Press, 2012).

19. Hwang, E. H. & Das Sarma, S. Dielectric function, screening, and plasmons in two-dimensional graphene. *Phys. Rev. B* **75,** 205418 (2007).

20. Mori, N. & Ando, T. Magnetophonon Resonance in Monolayer Graphene. *J. Phys. Soc. Japan* **80,** 044706 (2011).

21. Koshino, M. & Ando, T. Diamagnetism in disordered graphene. *Phys. Rev. B* **75,** 235333 (2007).

22. Yang, C. H., Peeters, F. M. & Xu, W. Density of states and magneto-optical conductivity of graphene in a perpendicular magnetic field. *Phys. Rev. B* **82,** 205428 (2010).
